# Interleukin-17 Cytokines and Receptors: Potential Amplifiers of Tendon Inflammation

**DOI:** 10.3389/fbioe.2021.795830

**Published:** 2021-12-23

**Authors:** Jolet Y. Mimpen, Sarah J. B. Snelling, Andrew J. Carr, Stephanie G. Dakin

**Affiliations:** The Botnar Research Centre, Nuffield Department of Orthopaedics, Rheumatology and Musculoskeletal Sciences, Medical Sciences Division, University of Oxford, Oxford, United Kingdom

**Keywords:** tendinopathy, interleukin-17, interleukin-17 receptor, inflammation, TNF—α, synergy

## Abstract

Interleukin (IL)-17A, a pro-inflammatory cytokine that is linked to the pathology of several inflammatory diseases, has been shown to be upregulated in early human tendinopathy and to mediate inflammatory and tissue remodelling events. However, it remains unclear which cells in tendons can respond to IL-17A, and how IL-17A, and its family members IL-17F and IL-17AF, can affect intracellular signalling activation and mRNA expression in healthy and diseased tendon-derived fibroblasts. Using well-phenotyped human tendon samples, we show that IL-17A and its receptors IL-17RA and IL-17RC are present in healthy hamstring, and tendinopathic and torn supraspinatus tendon tissue. Next, we investigated the effects of IL-17A, IL-17F, or IL-17AF on cultured patient-derived healthy and diseased tendon-derived fibroblasts. In these experiments, IL-17A treatment significantly upregulated *IL6*, *MMP3*, and *PDPN* mRNA expression in diseased tendon-derived fibroblasts. IL-17AF treatment induced moderate increases in these target genes, while little change was observed with IL-17F. These trends were reflected in the activation of intracellular signalling proteins p38 and NF- κ B p65, which were significantly increased by IL-17A, modestly increased by IL-17AF, and not increased by IL-17F. In combination with TNF-α, all three IL-17 cytokines induced *IL6* and *MMP3* mRNA expression to similar levels. Therefore, this study confirms that healthy and diseased tendon-derived fibroblasts are responsive to IL-17 cytokines and that IL-17A induces the most profound intracellular signalling activation and mRNA expression of inflammatory genes, followed by IL-17AF, and finally IL-17F. The ability of IL-17 cytokines to induce a direct response and activate diverse pro-inflammatory signalling pathways through synergy with other inflammatory mediators suggests a role for IL-17 family members as amplifiers of tendon inflammation and as potential therapeutic targets in tendinopathy.

## 1 Introduction

Tendinopathy (tendon pathology) is a common debilitating musculoskeletal disease. The aetiology is complex and multifactorial, disease is characterised by pain, a decline in function and reduced exercise tolerance (Maffulli et al., 1998). The pathophysiology of tendinopathy is hypothesised to be influenced by aging, repetitive use, genetic factors, and inflammation (Millar et al., 2017). However, the exact involvement of most of these mechanisms remains unknown. In this paper, we have defined tendinopathy as a multifactorial spectrum of tendon disorders that causes the tendon to become inflamed, and can ultimately lead to structural tissue damage (e.g., tendon tear). Although many therapies are used to treat tendinopathy, current treatments are ineffective and not evidence-based, reflecting the lack of understanding of the mechanisms underlying the pathophysiology (Nourissat et al., 2015).

The role of inflammation in tendinopathy has been controversial, but modern molecular and cellular techniques have shown the presence of immune cells and the activation of inflammatory pathways in tendinopathic tendons (Millar et al., 2017). Recent work shows that tendon stromal cells have an activated phenotype in disease, exemplified by the increased expression of stromal fibroblast activation markers such as podoplanin (PDPN), and that the interplay between these tendon stromal cells and immune cell populations may be a key driver of chronic tendon inflammation (Dakin et al., 2015; Dakin et al., 2017). One of the potential pro-inflammatory cytokines implicated in tendon inflammation is interleukin-17 (IL-17A), which was shown to be upregulated in early human tendinopathic tissue compared to matched controls and was able to mediate inflammation and tissue remodelling events (Millar et al., 2016). However, the role of the IL-17A pathway and its contribution to tendinopathy remains largely understudied.

IL-17 is a family of six pro-inflammatory cytokines IL-17A-F. The most well-studied family members, IL-17A and IL-17F, can function as homodimers or as the heterodimer IL-17AF. IL-17A and IL-17F have critical roles in host defence fighting bacterial and fungal infection (McGeachy et al., 2019). However, the pro-inflammatory qualities of these cytokines have made them important in the pathology of several chronic autoimmune and fibrotic inflammatory diseases (Miossec and Kolls, 2012), including psoriatic arthritis and ankylosing spondylitis, in which antibodies against IL-17A have revolutionised disease treatment (Boyd and Kavanaugh, 2015; Baraliakos et al., 2017). IL-17A, IL-17F, and IL-17AF all mainly act through a receptor complex comprised of IL-17 receptor A (IL-17RA) and IL-17RC (Kuestner et al., 2007; Wright et al., 2008). However, recent research has shown that IL-17A can signal through the IL-17RA/IL-17RD heterometric complex and that IL-17F might be able to signal through the IL-17RC/IL-17RC homodimeric complex (Su et al., 2019; Goepfert et al., 2020). IL-17RA is particularly highly expressed on immune cells, but is reported to be expressed by almost every cell type of the body. In contrast, IL-17RC is mostly expressed by non-immune cells, which mainly limits IL-17 signalling to non-hematopoietic epithelial and stromal cells (Yao et al., 1995; Kuestner et al., 2007). Although IL-17A is often reported to be the most potent cytokine followed by IL-17AF and finally IL-17F, several studies have shown that IL-17-induced effects differ between cell types. It remains unclear which cells in tendons can respond to IL-17A, and how IL-17A, IL-17F, and IL-17AF, can affect the intracellular signalling activation and mRNA expression in healthy and diseased tendon-derived stromal cells. To address this, we determined if IL-17 family receptors were expressed in healthy and tendinopathic human tendons, and to investigate the effects of IL-17A, IL-17F and IL-17AF treatments on cultured human tendon-derived fibroblasts.

## 2 Materials and Methods

### 2.1 Ethics

Ethical approval was granted for the Oxford Musculoskeletal Biobank (09/H0606/11) and (19/SC/0134) by the local research ethics committee (Oxford Research Ethics Committee B) for all work on human tendon, and informed consent was obtained from all patients according to the Declaration of Helsinki.

### 2.2 Tendon Tissue Acquisition

Healthy hamstring tendons were obtained from patients undergoing ACL reconstruction surgery and supraspinatus tendons from patients undergoing subacromial decompression surgery for early-stage pre-treatment tendinopathic supraspinatus through arthroscopic punch or from patients undergoing rotator cuff tear repair surgery. Tendon tear sizes were classified as small (≤1 cm), medium (>1 and ≤3 m), large (>3 and ≤5 cm) and massive (>5 cm in anterior-posterior length) (Post et al., 1983).

### 2.3 Immunohistochemistry

#### 2.3.1 Immunohistochemistry Staining and Imaging

Tendon samples were immersed in 10% formalin for 0.5 mm/h, embedded in paraffin before cutting 5 μm sections and baking onto adhesive glass slides. On the day prior to staining, tendon sections were baked at 60°C for 60 min and left at room temperature overnight to cool down. Deparaffinisation and antigen retrieval procedure was performed using a PT Link machine (Dako, Glostrup, Demark) using FLEX TRS antigen retrieval fluid (Dako). Immunostaining was performed using an Autostainer Link 48 machine using the EnVision FLEX visualisation system (Dako) with anti-human IL-17RA, anti-human IL-17RC antibodies (R&D systems, Abingdon, United Kingdom) or universal negative control mouse (Dako) (Supplementary Table S1). Antibody binding was visualized by FLEX 3,3′-diaminobenzidine (DAB) substrate working solution and haematoxylin counterstain (Dako) following the protocols provided by the manufacturer. Antibodies were validated in-house to determine the concentration of antibody needed for positive staining with minimal artifact from the tissue. After staining, slides were dehydrated before mounting using Pertex mounting medium (Histolab, Gothenburg, Sweden). Tissue sections were imaged using an Olympus BX51 inverted microscope (Olympus, Southend-on-Sea, United Kingdom) using DPManager and DPController software (Olympus) at 40x magnification. Negative controls are provided in Supplementary Figure S1.

#### 2.3.2 Immunofluorescence Staining

On the day prior to staining, tendon sections were baked at 60°C for 60 min and left at room temperature overnight to cool down. Deparaffinisation and antigen retrieval procedure was performed using a PT Link machine (Dako, Glostrup, Demark) by submerging slides in FLEX TRS antigen retrieval fluid.

Immunofluorescence staining was performed as published by Dakin et al. (2015). Briefly, after antigen retrieval, slides were left in buffer for 5 min, after which an ImmEdge pen (Thermo Fisher Scientific) was used to draw around the section to be stained. Specified sections were blocked for 1 h in PBS containing 5% normal goat serum (Sigma) in a humid chamber. After blocking solution was removed, sections were incubated with primary antibody (Supplementary Table S1) in PBS containing 5% normal goat serum (Sigma) and 0.05% saponin (Sigma) for 2 h. Sections were washed three times in PBS-T (0.2% Tween 20), and incubated in secondary antibody cocktail (Supplementary Table S2) in PBS with 5% equine serum and 0.05% saponin for 2 h in a humid chamber shielded from the light. After incubation, sections were washed three times with PBS-T and incubated with 2 mM POPO-1 nuclear counterstain (Invitrogen) diluted in PBS with 0.05% saponin for 20 min. After three washes in PBS-T, autofluorescence of the tissue was quenched using a 0.1% Sudan Black B solution (Applichem, Darmstadt, Germany) in 70% ethanol for 5 min. Sections were then washed three times with PBS-T before being mounted using fluorescent mounting medium (VectaShield, Vector laboratories, Peterborough, United Kingdom), sealed with nail polish, and stored at 4°C until image acquisition. Tendon tissue that was stained with universal mouse negative control antibody is shown in Supplementary Figure S2.

#### 2.3.3 Immunofluorescence Image Acquisition

Images for the immunofluorescence staining were acquired on a Zeiss LSM 710 confocal microscope (Carl Zeiss AG, Oberkochen, Germany) using a 40x objective with oil immersion (numerical aperture, 0.95). The fluorophores POPO-1, Alexa Fluor 488, Alexa Fluor 568, and Alexa Fluor 633 were excited with lasers lines at 405, 488, 561, and 633 nm, respectively. All channels were acquired sequentially to minimise bleed-through. Averaging was set to 2 and the pinhole was set to ±1 airy unit. ZEN 2009 software (Zeiss) was used to reconstruct two-dimensional images.

### 2.4 Isolation of Primary Tendon-Derived Fibroblasts for *in vitro* Culture

Healthy hamstring tendons and supraspinatus tendons with large or massive tears were used to isolate tendon-derived fibroblasts. Tendons were cut into ∼2 mm^3^ explants and incubated in DMEM-F12 media (Gibco, supplied by Fisher Scientific, Loughborough, United Kingdom) containing 50% Fetal Bovine Serum (FBS; Labtech International, Heathfield, United Kingdom) and 1% Penicillin/Streptomycin (P/S) (Gibco). Media was replaced twice a week, and cells were allowed to grow out from explants. Once cells were confluent, explants were removed and media replaced with D10 media (DMEM-F12 media supplemented with 10% FBS and 1% P/S). Cells were used at passage 3.

### 2.5 Treatment of Tendon-Derived Fibroblasts With IL-17 And/or TNF-α

Once tendon-derived fibroblasts at passage three were confluent, 30,000 cells were seeded in 24-well plates in D10 media and left for 24 h. Media was then replaced with serum-free media (DMEM-F12 with 1% P/S) to reduce background noise and synchronise the cell cycle, and cells were left for 24 h. On the day of the experiment, cytokines were made up in serum-free media [10 ng/ml IL-17A, IL-17F, and IL-17AF; 5 ng/ml TNF-α; or combination of IL-17 and TNF-α (all Biolegend United Kingdom Ltd., London, United Kingdom)] and added to the cells; vehicle controls were used for all cytokines. For mRNA, cells were harvested after 24 h using Trizol and stored at −80°C until further analysis. For proteins, cells were harvested after 0 min (baseline control), 10 min, 30 min, 1 h, or 8 h in lysis buffer at −20°C until further analysis (Morita et al., 2019).

### 2.6 RNA Extraction and RT-qPCR

RNA was extracted using the Direct-zol MicroPrep kit with DNase treatment (Zymo Research, Irvine, CA, United States) following the manufacturer’s instructions. RNA content was measured in eluted samples using a NanoDrop Machine (Implen GmbH, München, Germany). RNA was reverse transcribed into cDNA using the High Capacity Reverse Transcription Kit (Applied Biosystems). 10 μL real-time quantitative polymerase chain reaction (RT-qPCR) samples were prepared using 5 ng RNA, primers (Supplementary Table S3), and Fast SYBR Green Master Mix (Applied Biosystems, Foster City, California, United States) in 384 well plates, and performed using a ViiA7 (Life Technologies, Paisley, United Kingdom). Samples were analysed using the delta-delta Ct method using both Glyceraldehyde 3-phosphate dehydrogenase (*GAPDH*) and β-actin (*ACTB*) as reference genes.

### 2.7 SDS-PAGE and Western Blot

Cell lysates were mixed with 2x Laemmli Sample Buffer (Bio-Rad) in a 1-to-1 ratio and proteins were separated by gel electrophoresis in 10% Mini-PROTEAN TGX Precast gels (Bio-Rad Watford, United Kingdom). After gel electrophoresis, proteins were blotted on PVDF membrane (Bio-Rad) using a Trans-Blot Turbo Transfer System (Bio-Rad). Membranes were blocked in blocking buffer (10% milk powder and 2% BSA in TBS-T) and subsequently stained with loading control (vinculin) and phosphorylated-protein antibody overnight in antibody buffer (5% BSA and 1% Tween-20 in TBS-T) (Supplementary Table S4). The blots were washed in TBS-T and incubated in secondary antibody in antibody buffer for 2 h before washing again in TBS-T before visualising with ECL (GE Healthcare, Chicago, IL, United States) and imaging in an ALLIANCE 6.7 Chemiluminescence Imaging System (UVITEC, Cambridge, United Kingdom). The membranes were subsequently stripped (Takara BioInc, Kusatsu, Japan), blocked, and stained with total-protein antibody in antibody buffer overnight. Blots were then washed, stained with secondary antibody, washed again, and imaged. ImageJ was used to analyse the intensity of the stained bands for semi-quantitative analysis using the 0 min control for each patient as a reference. Supplementary Figure S3 shows representative western blots for each antibody.

### 2.8 Statistical Analysis

All statistical analyses were performed using in GraphPad Prism 9.2.0 (GraphPad Software, La Jolla, CA, United States). To study the differences between vehicle and IL-17 cytokine treatment at each time point of the western blot analysis, two-way ANOVA with Dunnett’s multiple comparison’s tests were used. For RT-qPCR experiments with IL-17 cytokines, differences between vehicle and IL-17 cytokine treatments were tested with Friedman test with Dunn’s test to correct for multiple comparisons. * = *p* < 0.05, ** = *p* < 0.01, *** = *p* < 0.001, **** = *p* < 0.0001.

## 3 Results

### 3.1 Cells Derived From Healthy and Diseased Tendons Are Responsive to IL-17 Cytokines

**FIGURE 1:**
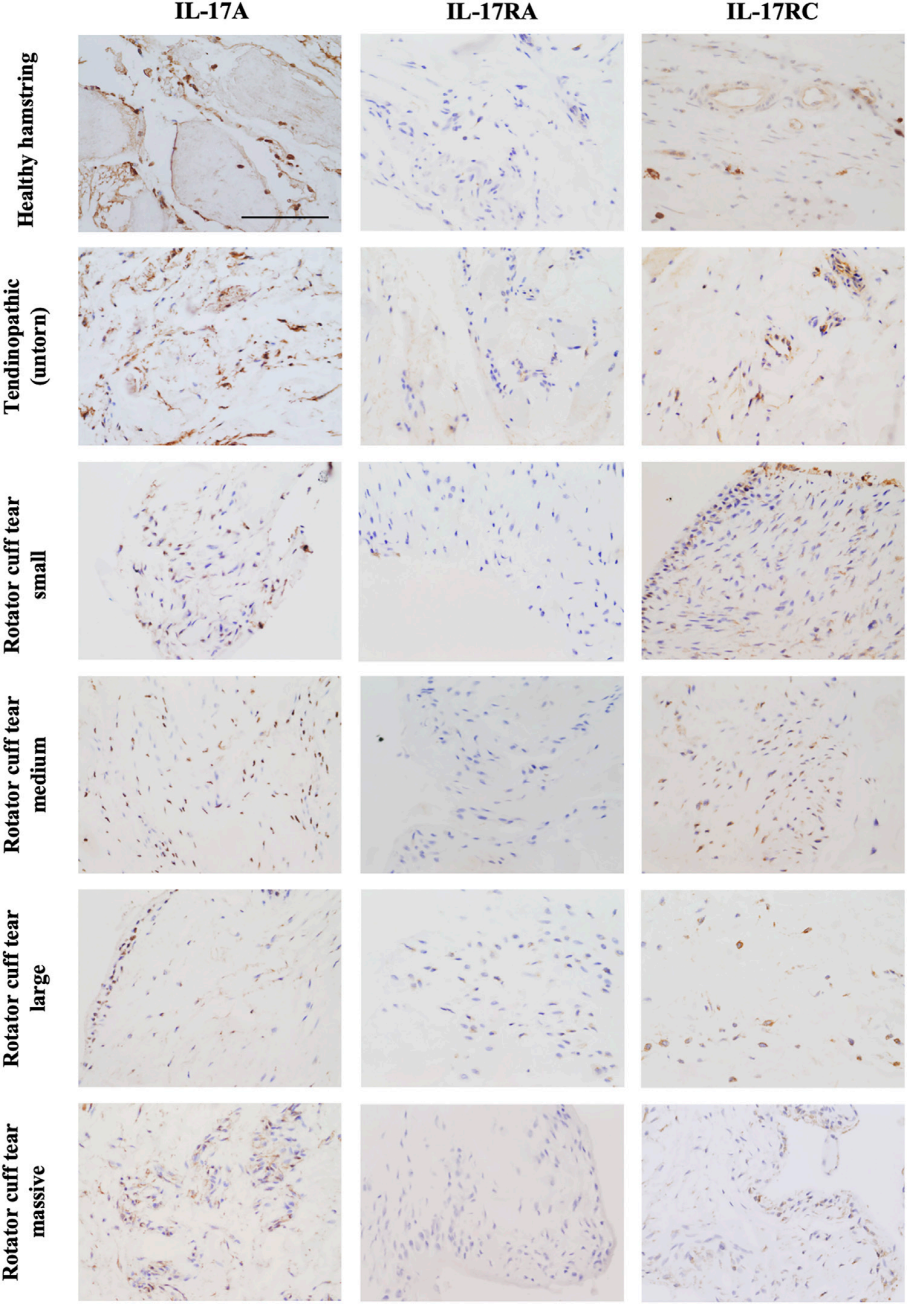
Representative images showing protein expression of IL-17A and IL-17 receptor A (IL-17RA) and IL-17RC in healthy tendon tissue (hamstring tendon), or diseased tendon tissue ranging from tendinopathic (untorn) to small, medium, large, and massive rotator cuff tears (supraspinatus tendon). Antibodies were visualised with DAB (brown) and nuclei counterstained with haematoxylin (blue). Images were taken at 40x magnification; scale bar = 100 μm.

**FIGURE 2:**
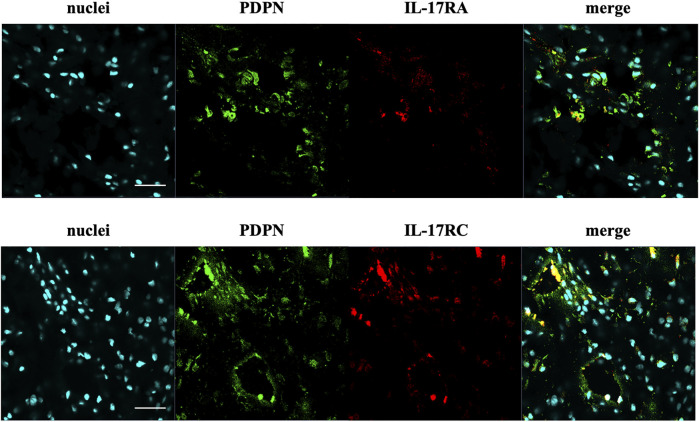
Representative images of the protein expression of podoplanin (PDPN, green) and IL-17RA or IL-17RC (red) in supraspinatus tendons with a large/massive tear. POPO-1 staining was used to visualise nuclei (cyan). Images taken at 40x; scale bar = 20 μm.

The presence of IL-17A protein was confirmed in healthy, tendinopathic (inflamed), and torn tendon tissues. (Figure 1). To determine if cells in healthy or diseased tendon tissue can respond to IL-17 cytokines, these tissues were stained for IL-17 receptors IL-17RA and IL-17RC. Whilst IL-17RC expression was abundant in healthy and diseased tendon tissues, only low levels of IL-17RA expression were found. Co-staining of PDPN, a marker for activated fibroblasts, and IL-17RA or IL-17RC in large/massive supraspinatus tear tissues revealed that IL-17 receptors seemed to be mainly expressed by PDPN+ cells (Figure 2). Expression of *IL17RA* and *IL17RC* mRNA was confirmed in tendon-derived fibroblasts from healthy hamstring and supraspinatus tendon with large or massive tears. *IL17RC* was more highly expressed than *IL17RA* in both cell types, and expression of both receptors was higher in fibroblasts derived from diseased than from healthy tendon (Supplementary Figure S4).

### 3.2 IL-17A is a More Potent Inducer of Pro-inflammatory Genes Than IL-17F and IL-17AF in Tendon-Derived Fibroblasts

**FIGURE 3:**
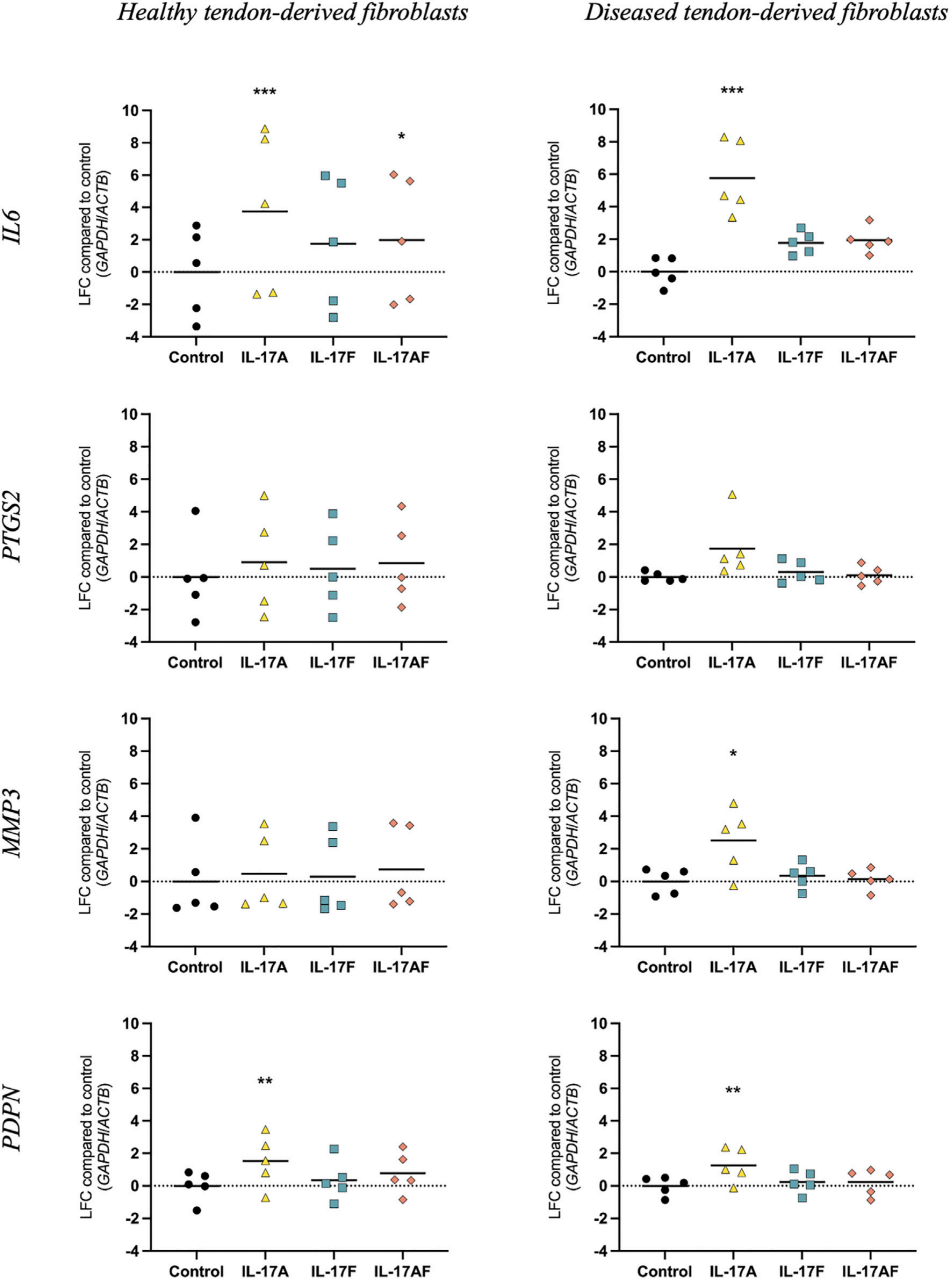
Changes in mRNA expression after treatment with IL-17A, IL-17F, or IL-17AF compared to vehicle control in fibroblasts derived from healthy hamstring or diseased supraspinatus tendons with a large or massive tears. mRNA expression was calculated using the ddCT-method using the reference genes *GAPDH* and *ACTB*, and is expressed as log2 fold change (LFC) compared to control. Changes in mRNA expression compared to control were calculated using a Friedman test with Dunn’s multiple comparisons test. Individual values and mean. *N* = 5. * = *p* < 0.05, ** = *p* < 0.01, *** = *p* < 0.001.

To study how different IL-17 cytokines affect mRNA expression of tendon-derived fibroblasts, tendon fibroblasts derived from healthy hamstring or large/massive supraspinatus tears (diseased) were treated with vehicle control, or 10 ng/ml IL-17A, IL-17F, or IL-17AF for 24 h and mRNA expression was assessed using RT-qPCR (Figure 3). IL-17A treatment induced a significant increase in mRNA expression of *IL6* and *PDPN* in healthy tendon-derived fibroblasts and of *IL6*, *MMP3*, and *PDPN* in diseased tendon-derived fibroblasts. While IL-17AF, and to a lower extent IL-17F, seemed to induce slight increases in *IL6*, with IL-17AF reaching significance in healthy tendon-derived fibroblasts, the mRNA expression changes observed for both IL-17AF and IL-17F were notably lower than IL-17A. While IL-17 cytokines induced similar mRNA expression changes of *IL6*, *PTGS2*, and *PDPN* in healthy and diseased tendon-derived fibroblasts, the expression of *MMP3* was only significantly increased by IL-17A in diseased tendon-derived fibroblasts, while no change was found in healthy tendon-derived fibroblasts. These findings suggest that fibroblasts derived from diseased tendons may respond differently to IL-17 family cytokines than fibroblasts derived from healthy tendons.

### 3.3 IL-17A Induces Activation of p38 and NF-κB Pathways

**FIGURE 4:**
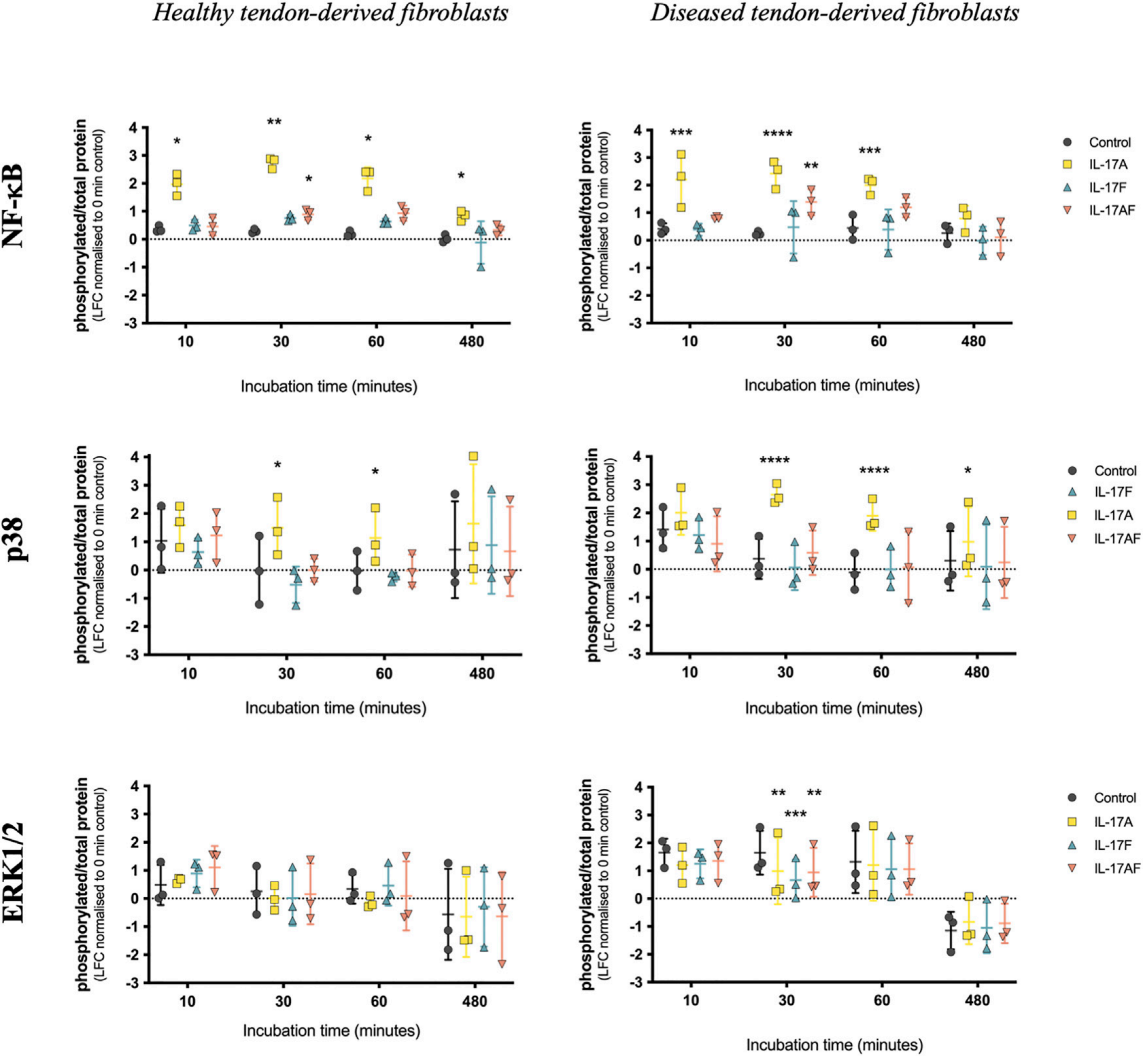
Activation of p65 NF-κB, p38, and ERK1/2, after stimulation with 10 ng/ml IL-17A, IL-17F, or IL-17AF in tendon fibroblasts derived from healthy hamstring or supraspinatus tendons with large or massive tears. Phosphorylated protein over total protein ratios compared to baseline control (0 min). Two-way ANOVA with Dunnett’s multiple comparison’s test. Mean ± SD. *N* = 3. * = *p* < 0.05, ** = *p* < 0.01, *** = *p* < 0.001, **** = *p* < 0.0001.

To better understand the differences and similarities of the different IL-17 cytokines in the activation of intracellular signalling pathways in healthy and diseased human-derived tendon cells, western blots were used to assess the activation of intracellular signalling proteins ERK1/2 (p44/42 MAP kinases), p38, and NF-κB p65. Healthy or diseased human tendon-derived fibroblasts were treated with vehicle control or 10 ng/ml IL-17A, IL-17F, or IL-17AF for 10, 30 min, 1or 8 h (Figure 4); semi-quantitative analysis was carried out using a 0 h control as the reference. IL-17A induced a strong increase in NF-κB p65 activation at all time points in healthy tendon-derived fibroblasts, and after 10 min, 30 min, and 1 h in diseased tendon-derived fibroblasts. IL-17AF induced a significant increase in activation after 30 min in both cell types, while the small increases caused by IL-17F were not statistically significant. Activation of p38 by IL-17A reached significance after 30 min and 1 h incubation in healthy tendon-derived fibroblasts and after 30 min, 1 h, and 8 h in diseased tendon-derived fibroblasts. IL-17AF and IL-17F did not cause any increases in p38 activation. No significant increases in activation of ERK1/2 were found for any of the IL-17 cytokines in either cell type. However, all IL-17 cytokines led to a significant decrease in activation after 30 min incubation in diseased tendon-derived fibroblasts.

### 3.4 IL-17 Cytokines Synergise With TNF-α

**FIGURE 5:**
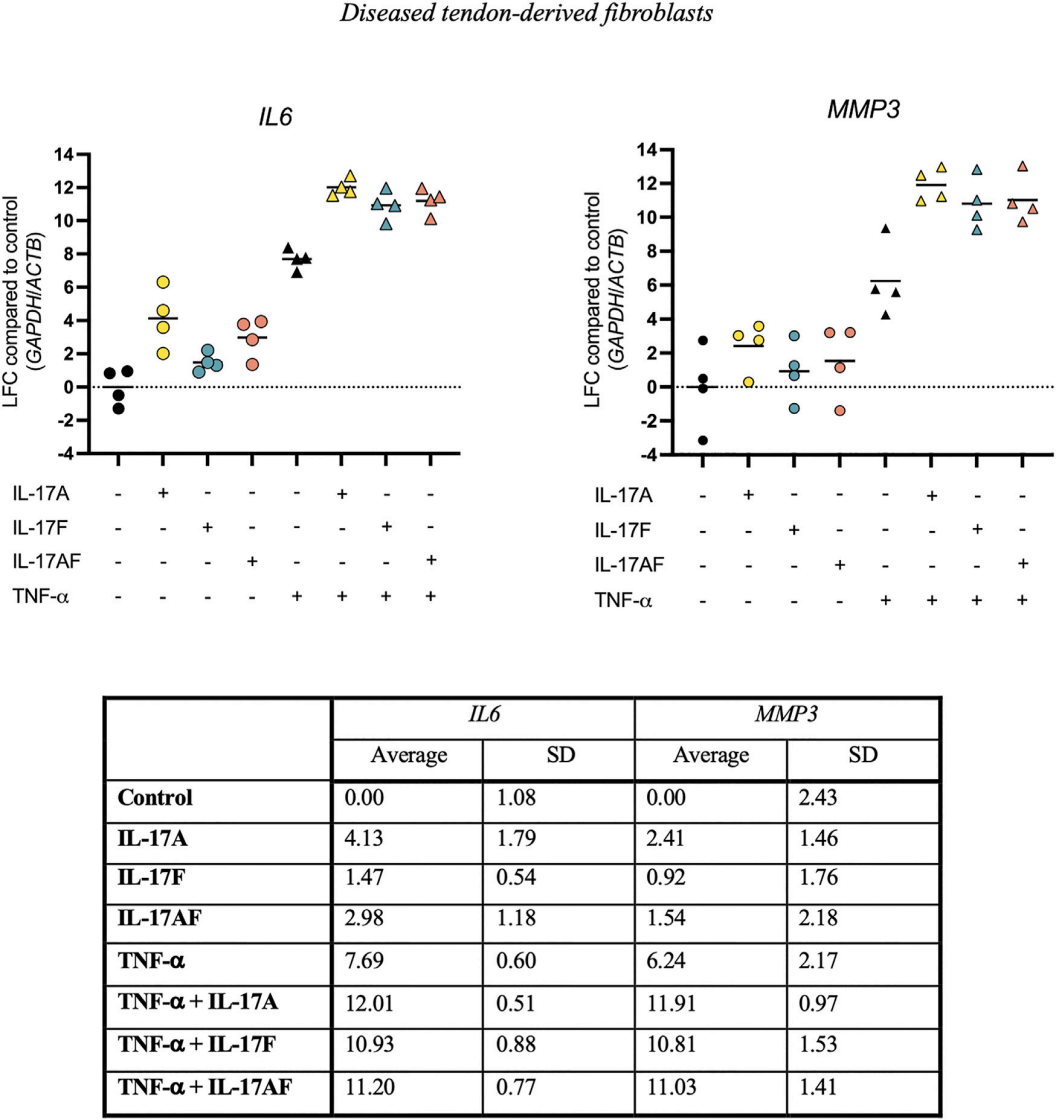
Changes in *IL6* and *MMP3* mRNA expression after treatment with IL-17A, IL-17F, or IL-17AF alone or in combination with TNF-α in fibroblasts derived from diseased supraspinatus tendons with large or massive tears. mRNA expression was calculated using the ddCT-method using the reference genes *GAPDH* and *ACTB*, and is expressed as log2 fold change (LFC) compared to control. Individual values and mean. *N* = 4.

To test the ability of IL-17 cytokines to synergise with other cytokines, tendon fibroblasts derived from large or massive supraspinatus tears were treated with IL-17 cytokines alone or in combination with TNF-α for 24 h. Expression of *IL6* and *MMP3* mRNA was assessed using RT-qPCR. Although IL-17A alone caused a much stronger increase in *IL6* expression (LFC 4.13) than IL-17F (LFC 1.47) or IL-17AF (LFC 2.98) alone, in combination with TNF-α, IL-17A, IL-17F, and IL-17AF all caused a similar increase in mRNA expression (LFC 12.01, 10.93, and 11.20, respectively) (Figure 5). Although the differences between the three IL-17 cytokines alone was smaller in *MMP3*, all three IL-17 cytokines also caused a similar increase in *MMP3* expression in combination with TNF-α (LFC 11.91, 10.81, and 11.03, respectively). Interestingly, the increase in *IL6* and especially *MMP3* mRNA expression after treatment with TNF-α and IL-17 cytokines together was higher than TNF-α alone, IL-17 alone, or the effect of TNF-α alone and IL-17 alone combined, suggesting a synergistic effect.

## 4 Discussion

IL-17 cytokines have been implicated in the pathophysiology of several inflammatory musculoskeletal diseases, including ankylosing spondylitis and psoriatic arthritis. A recent study has shown that IL-17A was upregulated in early human tendinopathic tissue compared to matched controls and could mediate inflammatory and tissue remodelling events (Millar et al., 2016). However, the IL-17A pathway remains largely underexplored in tendinopathy. Therefore, the aim of this study was to study the expression of IL-17 receptors in healthy, tendinopathic, and torn tendons, and to compare the activity and function of IL-17A, IL-17F and IL-17AF in human tendon-derived fibroblasts.

Protein expression of IL-17A and protein and mRNA expression IL-17RC was detected in tendon tissue from both healthy and diseased tissue, while only low levels of IL-17RA protein and mRNA expression were found. In diseased supraspinatus tendon, IL-17 receptors seemed to mainly be present on cells that stained positive for PDPN (a marker for activated fibroblasts), which suggests that activated fibroblasts are the predominant cells that are responsive to IL-17 signalling in tendon. However, expression of IL-17RA seems to be the limiting factor for IL-17 signalling in tendon. More research is needed to further identify which cells in healthy and diseased tendons are responsive to IL-17 signalling and which cells in tendons are responsible for the production of IL-17 cytokines.

When comparing the effect of IL-17 cytokines IL-17A, IL-17F, and IL-17AF on the mRNA expression of *IL6*, *PTGS2*, *MMP3*, and *PDPN* in tendon-derived fibroblasts from healthy or diseased tendons, IL-17A induced the most potent changes, followed by IL-17AF, and finally IL-17F. No striking differences were seen between healthy and diseased tendon-derived fibroblasts in the IL-17-induced mRNA expression of *IL6*, *PTGS2*, and *PDPN*. However, *MMP3* was significantly increased after IL-17A treatment in diseased tendon-derived fibroblasts, while no significant change was seen in healthy tendon-derived fibroblasts. The differences in potency between the three IL-17 cytokines are reflected in the western blot data, which shows the strongest increases in intracellular signalling are caused by IL-17A, followed by IL-17AF, and finally IL-17F. In addition, there seems to be a stronger increase in activation of p38 signalling after IL-17A treatment in tendon fibroblasts derived from diseased tendon compared to those derived from healthy tendon, which supports the difference seen in *MMP3* mRNA expression. Therefore, tendon fibroblasts derived from diseased tissue might respond differently to IL-17 cytokines than tendon fibroblasts derived from healthy tissue; a phenomenon that has been shown previously with other mediators (Dakin et al., 2015). Interestingly, dysregulated extracellular matrix turnover, which is thought to be due to changes in the metabolic turnover of macromolecules, has been implied in the pathophysiology of tendinopathy (Samiric et al., 2009; Millar et al., 2021). Therefore, IL-17-induced increases in *MMP3*, which encodes for the MMP-3 enzyme that degrades several types of collagens, proteoglycans, as well as other extracellular matrix macromolecules, could contribute to tendinopathy. However, more research needs to be to look at the effects of IL-17 cytokines across the whole transcriptome and on protein level to better understand IL-17-induced changes in tendon-derived fibroblasts and the potential role of IL-17 cytokines in tendon disease.

Due to the complex nature of the inflammatory environment during tendon disease, cytokines should be studied in combination with other cytokines. This study showed that all three IL-17 cytokines in combination with TNF-α can synergistically induce the expression of *IL6* and *MMP3* mRNA expression in tendon fibroblasts derived from diseased tissue; a phenomenon that has been observed in several other cell types previously (Gaffen, 2004; McGeachy et al., 2019). Interestingly, treatment of TNF-α in combination with IL-17F and IL-17AF induced a similar increase in mRNA expression as TNF-α in combination with IL-17A, which is much more potent than IL-17F and IL-17AF by itself. This ability to amplify biological signals, including inflammatory signals, could be a critical function of IL-17 in tendon disease.

IL-17 cytokines have important roles in innate immunity, as they protect against fungal and bacterial infections, and have a role in tissue healing (Lai et al., 2012; Lee et al., 2015; Maxwell et al., 2015; Bunte and Beikler, 2019; Chen et al., 2019), but these host-protective functions can also cause give rise to immunopathology in autoimmunity, cancer, and other inflammatory diseases (McGeachy et al., 2019). Veldhoen (2017) hypothesizes that IL-17 mediated recruitment of immune cells might be able to contribute to the initiation of chronic inflammation and autoimmunity, but that IL-17 might not be essential to sustain chronic inflammation (Veldhoen, 2017)*.* Although this study and the previous study by Millar et al. have shown that IL-17 cytokines can induce certain effects in tendon cells (Millar et al., 2016), it is important to establish if high enough levels of IL-17 are present in disease to contribute to the disease initiation or maintenance, and if so, what the role of IL-17 cytokines is. Future studies should investigate at which levels IL-17 is present in health and at different stages of disease, and what the source of these cytokines is. In addition, as it has been shown that IL-17-induced effects differ between cell types (Ha et al., 2014; McGeachy et al., 2019), it is important to look at the effects of IL-17 in tendon across the whole transcriptome or proteome to better understand which pathways IL-17 cytokines could affect in tendon-derived fibroblasts at different stages of disease.

In conclusion, IL-17A and its receptors are present in human healthy and diseased tendon tissues, although IL-17RC protein expression is much more abundant than expression of IL-17RA. IL-17A is the most potent cytokine in activation of intracellular signalling pathways p38 and NF-κB p65 and mRNA expression of genes including *IL6*, *MMP3*, and *PDPN*, followed by IL-17AF, and finally IL-17F. However, in combination with TNF-α, all three IL-17 cytokines induce similar levels of *IL6* and *MMP3* mRNA expression, which could make not only IL-17A, but also IL-17F and IL-17AF interesting treatment targets in tendinopathy. Collectively these findings suggest a role for IL-17 family members as amplifiers of tendon inflammation and as potential therapeutic targets in tendinopathy.

## Data Availability

The raw data supporting the conclusion of this article will be made available by the authors, without undue reservation.
