# Supplementary material for: Interleukin-17 Cytokines and Receptors: Potential Amplifiers of Tendon Inflammation
Source: Front Bioeng Biotechnol. 2021 Dec 23;9:795830. doi: 10.3389/fbioe.2021.795830 (PMC8733930; doi:10.3389/fbioe.2021.795830)
Supplement: Supplementary file 1 [file DataSheet1.PDF]

<u>Supplementary Material</u>

Interleukin-17 cytokines and receptors: potential amplifiers of tendon inflammation

Jolet Y. Mimpen, Andrew J. Carr, Sarah J.B. Snelling, Stephanie G. Dakin

Supplementary Figures

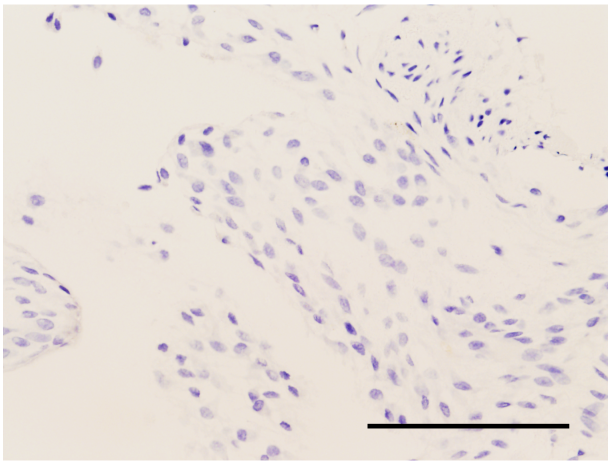

Supplementary Figure 1. Representative image showing isotype control using universal negative control mouse (Dako) on a section of supraspinatus tendon with a massive tear, counterstained with haematoxylin (blue). Images were taken at 40x magnification; scale bar = 100 μm.

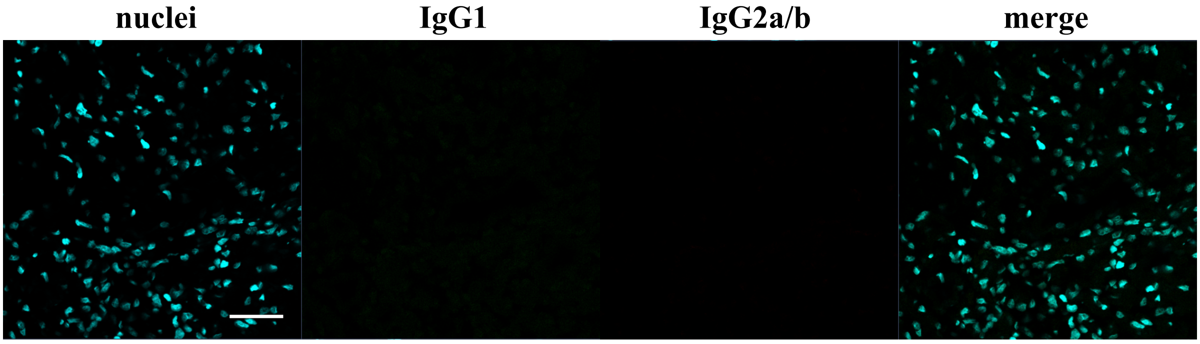

Supplementary Figure 2. Representative image showing isotype control staining using universal negative control mouse (IgG1, IgG2a, IgG2b) (Dako) on a section of supraspinatus tendon with a massive tear. Nuclei are counterstained with POPO-1 (cyan). Images taken at 40x; scale bar = 20 μm

Supplementary Figure 3A-C. Representative western blots that were incubated with antibodies for phosphorylated antibody and vinculin, washed, incubated with secondary antibody, and visualised with ECL. The blots were then stripped, blocked, and incubated with antibody for total protein, washed, incubated with secondary antibody, and visualised with ECL.

Supplementary Figure 3A. NF-κB.

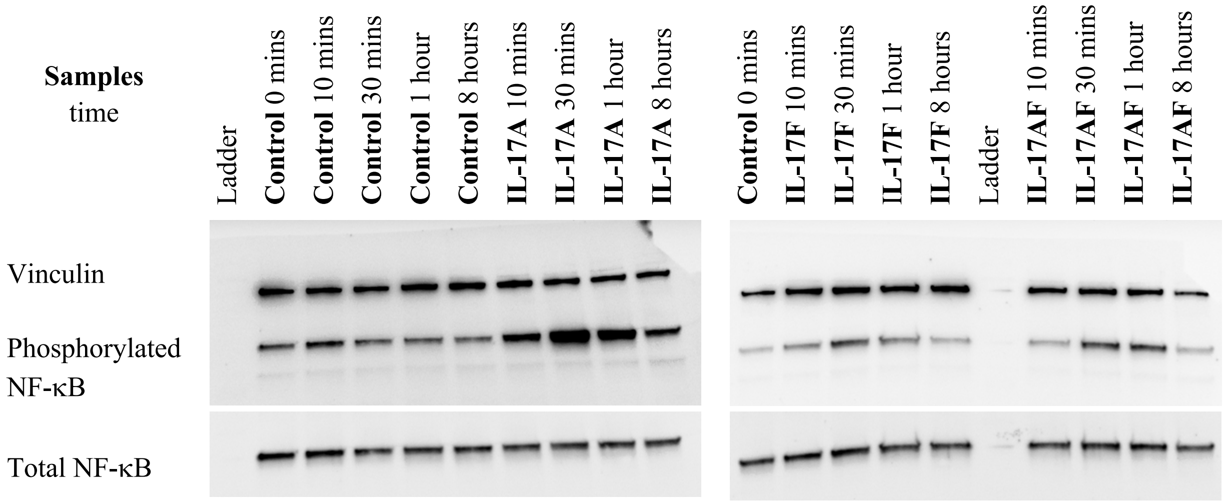

Supplementary Figure 3B. p38.

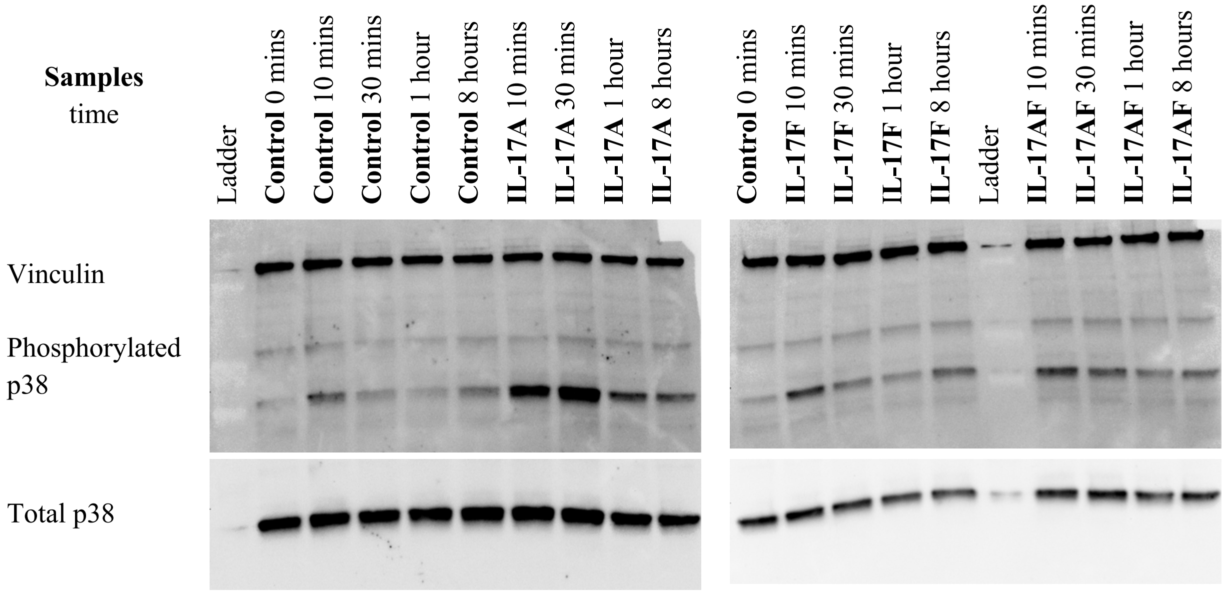

Supplementary Figure 3C. ERK1/2.

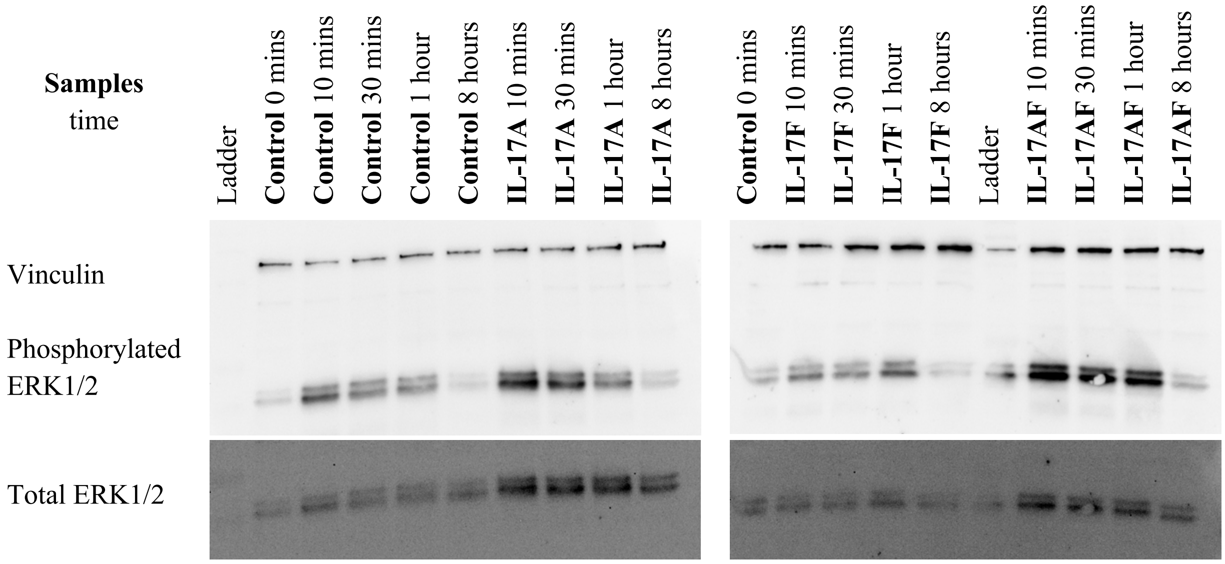

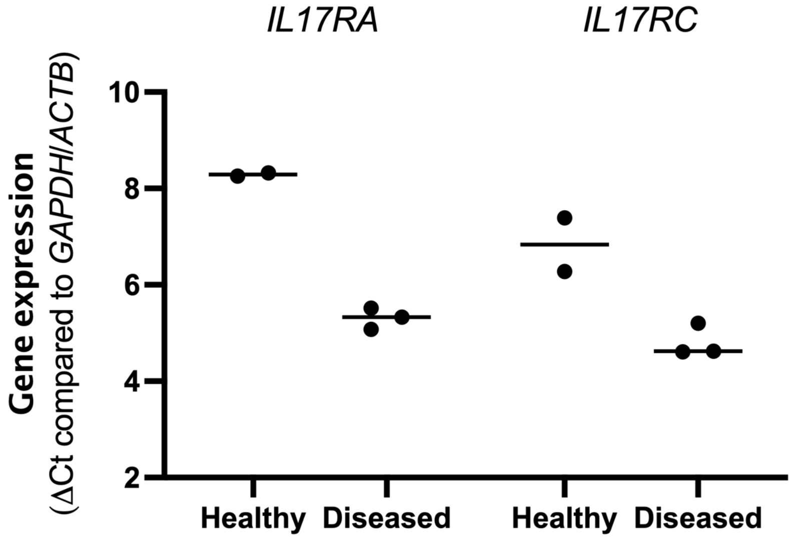

Supplementary Figure 4. *IL17RA* (left) and *IL17RC* (right) mRNA expression in human tendon-derived cells from healthy hamstring tendons (healthy) and supraspinatus tendons with a large or massive tear (diseased), showing a relatively higher mRNA expression of IL-17 receptors in diseased tissue compared to healthy comparator tissue. Individual values and mean. N=2 (hamstring), N=3 (large/massive supraspinatus tendon tears).

Supplementary Tables

Supplementary Table 1. Primary antibodies used for immunohistochemistry and immunofluorescence, including the antibody target, isotype, supplier, reference number, and dilution at which the antibody was used.

Target	Isotype	Supplier	Ref number	Dilution
Human IL-17A	Monoclonal mouse IgG1	ProteinTech Group Inc, Rosemont, IL, USA	66148-1-Ig	1:500
Human IL-17RA	Monoclonal mouse IgG2B	R&D systems	MAB1771	1:50
Human IL-17RC	Monoclonal mouse IgG2B	R&D	MAB22691	1:1000
Human podoplanin (PDPN)	Monoclonal mouse IgG1	Abcam	ab10288	1:200
Universal negative control mouse	Cocktail of mouse IgG1, IgG2a, IgG2b, IgG3, and IgM	Dako, Glostrup, Denmark	IR750	Ready to use

Supplementary Table 2. Secondary antibodies used for immunofluorescence, including antibody target, supplier, reference number, and dilution at which the antibody was used.

Target	Supplier	Ref number	Dilution
Goat anti-mouse IgG1 (Alexa Fluor 488)	Southern Biotech (supplied by Cambridge Bioscience Ltd, Cambridge, UK)	1070-02	1:200
Alexa Fluor goat anti-mouse IgG2b (γ2b)	Invitrogen	A21144	1:200

Supplementary Table 3. Primers used for RT-qPCR, including forward and reverse sequences.

Target	Supplier	Forward	Reverse
GAPDH	Designed in-house	GAAGGTTGAAGGTCG GAGTC	GAAGATGGTGATGGGAT TTC
ACTB	Designed in-house	CCTGGCACCCAGCAC AAT	GCCGATCCACACGGAGT ACT
IL6	Primerdesign Ltd	GCAGAAAACAACCTG AACCTT	ACCTCAAACTCCAAAAG ACCA
IL17RA	Primerdesign Ltd	TGAGCACATGCACCA CATAC	TTAAGGTTGCGTAGAGT GAGTG
IL17RC	Primerdesign Ltd	AGTCGTGCTCTCCTTC CAG	AGATTCGTACCTTCACT CCCTAG
MMP3	Primerdesign Ltd	ATGATGAACAATGGA CAAAGGATAC	AGTGTTGGCTGAGTGAA AGAG
PTGS2	Primerdesign Ltd	CAGGCTTCCATTGAC CAGAG	TTTCTCCTGTAAGTTCTT CAAATGAT
PDPN	Primerdesign Ltd	CCGAAGATGATGTGG TGACTC	CGATGCGAATGCCTGTT ACA

Supplementary Table 4. Primary and secondary antibodies used for western blot experiments, including target, supplier, reference number, and the dilution at which each antibody was used.

Target	Supplier	Ref number	Dilution
Vinculin (reference protein)	Cell Signalling Technology, Danvers, MA, USA	13901	1:1000
phospho-ERK1/2	Cell Signalling Technology	4370	1:2000
ERK1/2	Cell Signalling Technology	4695	1:1000
phospho-p65 NF-κB	Cell Signalling Technology	3033	1:1000
p65 NF-κB	Cell Signalling Technology	8242	1:1000
phospho-p38	Cell Signalling Technology	4511	1:1000
p38	Cell Signalling Technology	8690	1:1000
Anti-rabbit IgG, HRP-linked	Cell Signalling Technology	7074	1:1000 (phospho-antibodies) 1:2000 (total-antibodies)
